# Maternal smoking status before and during pregnancy and bronchial asthma at 3 years of age: a prospective cohort study

**DOI:** 10.1038/s41598-023-30304-9

**Published:** 2023-02-24

**Authors:** Kunio Miyake, Megumi Kushima, Ryoji Shinohara, Sayaka Horiuchi, Sanae Otawa, Yuka Akiyama, Tadao Ooka, Reiji Kojima, Hiroshi Yokomichi, Zentaro Yamagata, The Japan Environment and Children’s Study Group, Michihiro Kamijima, Shin Yamazaki, Yukihiro Ohya, Reiko Kishi, Nobuo Yaegashi, Koichi Hashimoto, Chisato Mori, Shuichi Ito, Zentaro Yamagata, Hidekuni Inadera, Takeo Nakayama, Hiroyasu Iso, Masayuki Shima, Youichi Kurozawa, Narufumi Suganuma, Koichi Kusuhara, Takahiko Katoh

**Affiliations:** 1 Department of Health Sciences, School of Medicine, University of Yamanashi, 1110 Shimokato, Chuo, Yamanashi 409-3898 Japan; 2 Center for Birth Cohort Studies, University of Yamanashi, Yamanashi, Japan; 3 Principal investigator, Nagoya City University, Nagoya, Japan; 4 National Institute for Environmental Studies, Tsukuba, Japan; 5 National Center for Child Health and Development, Tokyo, Japan; 6 Hokkaido University, Sapporo, Japan; 7 Tohoku University, Sendai, Japan; 8 Fukushima Medical University, Fukushima, Japan; 9 Chiba University, Chiba, Japan; 10 Yokohama City University, Yokohama, Japan; 11 University of Yamanashi, Chuo, Japan; 12 University of Toyama, Toyama, Japan; 13 Kyoto University, Kyoto, Japan; 14 Osaka University, Suita, Japan; 15 Hyogo College of Medicine, Nishinomiya, Japan; 16 Tottori University, Yonago, Japan; 17 Kochi University, Nankoku, Japan; 18 University of Occupational and Environmental Health, Kitakyushu, Japan; 19 Kumamoto University, Kumamoto, Japan

**Keywords:** Diseases, Risk factors

## Abstract

The association between maternal pre-pregnancy smoking status and asthma risk is unclear. This study aimed to investigate the association between pre- and post-pregnancy maternal smoking status and bronchial asthma at 3 years of age in a large birth cohort. Data of 75,411 mother–child pairs from the Japan Environment and Children's Study (JECS) were analysed using multivariate logistic regression analysis. Overall, 7.2% of the children had bronchial asthma. The maternal smoking status before childbirth was as follows: Never = 60.0%, Quit before recognising current pregnancy = 24.1%, Quit after finding out about current pregnancy = 12.3%, and Still smoking = 3.6%. Children of mothers who sustained smoking during pregnancy had an increased risk of bronchial asthma at 3 years of age even after adjusting for pre- and postnatal covariates (adjusted odds ratio [aOR] 1.34, 95% confidence interval [CI] 1.15–1.56). Children of mothers who quit before (aOR 1.09, 95% CI 1.02–1.18) or after (aOR 1.11, 95% CI 1.01–1.23) recognising the current pregnancy had an increased risk of bronchial asthma at 3 years of age. Maternal smoking throughout pregnancy and smoking exposure pre-pregnancy or in early pregnancy increases the risk of bronchial asthma in children.

## Introduction

Asthma is one of the most common illnesses in children^1^. The trend of the prevalence of asthma varies with country and region. The global incidence of asthma is increasing in low-income countries but is flat or decreasing in some developed countries^2^. The prevalence of asthma in Japanese schoolchildren has also been declining in recent years (6.5% in 2002 and 4.7% in 2012)^3^.

Several risk factors for asthma in children have been reported, including a history of parental asthma, prenatal smoking exposure, preterm birth, birth by caesarean delivery, indoor exposure to mould or fungi, and outdoor air pollution^4,5^. In contrast, breastfeeding and high dietary fruit intake during pregnancy have been reported to have a protective effect on asthma in children^6,7^. It is well known that maternal smoking exposure during pregnancy increases the risk of asthma in children^8–10^. In Japan, the smoking rate among adult women was reported to be 7.6% in 2019^11^, and that among pregnant women was 3.8% in 2013 and 2.9% in 2016^12^. Maternal second-hand smoke (SHS) exposure during pregnancy is also associated with an increased risk of asthma in children^13–15^. Regarding postnatal SHS exposure, five European birth cohort studies from the Mechanisms of the Development of Allergy reported that SHS exposure in infants was not statistically significant but was associated with an increased risk of asthma up to 14–16 years of age^16^.

There is controversy about the health effects on children from mothers who quit smoking before or early in pregnancy. A large-scale population-based retrospective cohort study using the United States National Vital Statistics System data from 2011 to 2018 found that the risk of preterm birth significantly increases even if the mother quits smoking during the first trimester^17^. In contrast, it has been reported that maternal smoking cessation before and early in pregnancy does not increase the risk of a small-for-gestational-age infant and childhood overweight at 3 years^18^. Regarding asthma in children, a retrospective hospital-based birth cohort study in Finland found that paternal smoking cessation during pregnancy was associated with a reduced risk of asthma in children, while the effect of maternal smoking cessation during pregnancy and the risk of asthma in children were unclear^14^.

Limitations of previous studies include recall bias in retrospective studies, differences in the definition of smoking environment, and the inability to adjust for essential confounding factors. Therefore, there is a need for verification using a large-scale prospective birth cohort. The purpose of this study was to clarify the association between the prenatal maternal smoking status and the risk of bronchial asthma at 3 years of age by adjusting for covariates before and after birth using a large cohort Japan Environment and Children's Study (JECS).

## Methods

### Study design and population

This study used data from JECS, a nationwide birth cohort study. JECS is a project aimed at investigating the effects of environmental factors on child health and development. The detailed protocols have been published elsewhere^19,20^. Fifteen Regional Centres across Japan participate in JECS, and women in early pregnancy and their partners who lived in the area around the Regional Centre were recruited and followed up by the Centre. Participants were recruited between January 2011 and March 2014. Participating children will be tracked until they reach the age of 13 years. The JECS protocol was reviewed and approved by the Ministry of the Environment’s Institutional Review Board on Epidemiological Studies and the Ethics Committees of all participating institutions (Ethical Number: No.100910001). JECS is conducted in accordance with the principles of the Declaration of Helsinki and with written informed consent from all participants.

**Figure 1:**
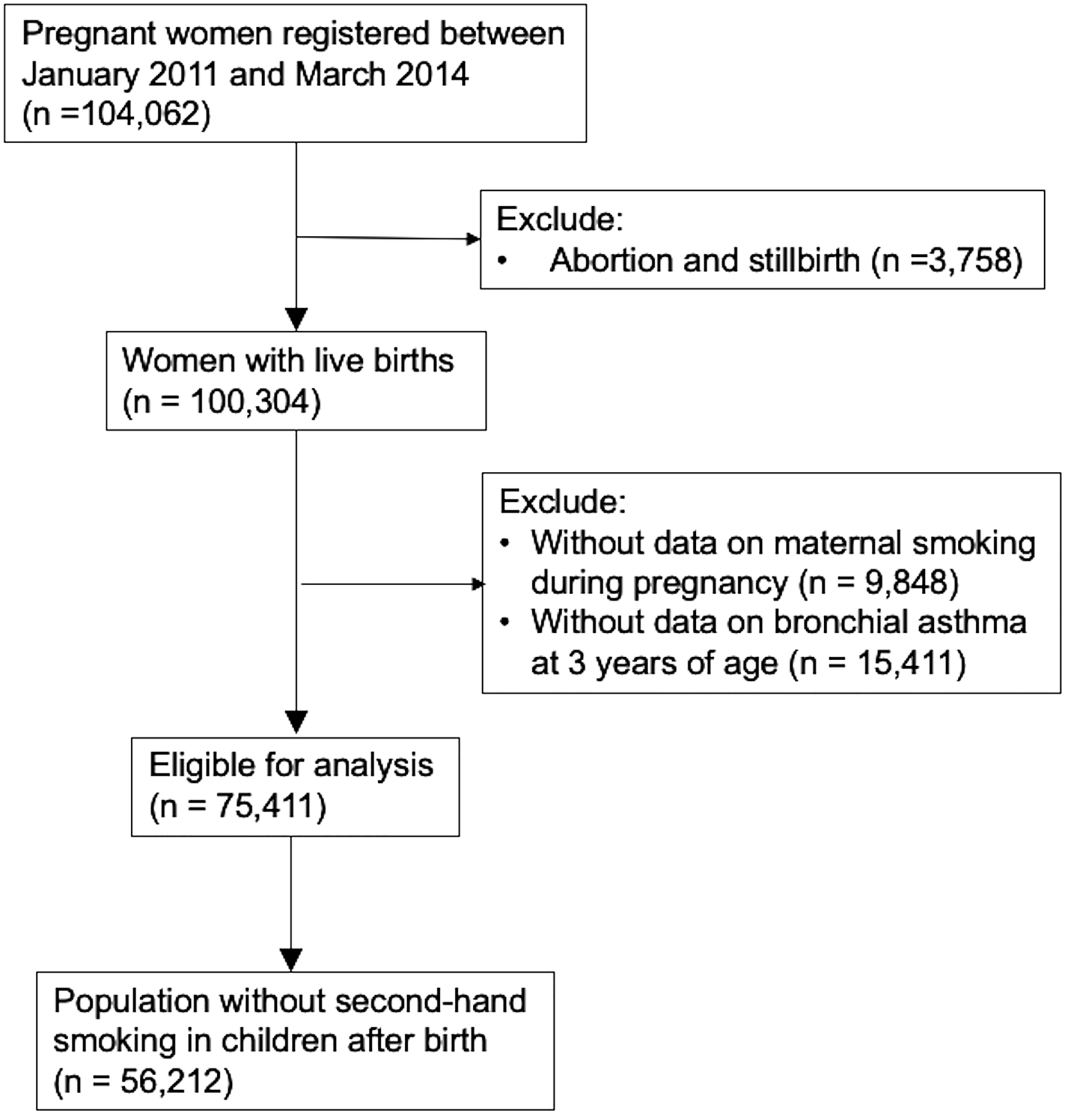
Flow chart of participant selection.

We used the dataset ‘Jecs-ta-20190930-qsn’ which was released in October 2019. Of the 100,304 live births, we excluded those with missing data on maternal smoking status before and after pregnancy and bronchial asthma at 3 years of age. Finally, 75,411 mother–child pairs were analysed in the present study (Fig. 1).

### Exposure and covariates

Data on prenatal parental smoking history and exposure to SHS were collected using a self-administered questionnaire during the second or third trimester of pregnancy. A mother was instructed to choose among 'Never'; 'Previously did, but quit before recognising current pregnancy'; 'Previously did, but quit after finding out about current pregnancy’; or ‘Yes, I still smoke’ respective to herself and her partner. We collected information from mothers with a history of smoking about the average number of cigarettes smoked per day, the age at starting smoking, and the age at quitting smoking. In addition, for mothers who quit smoking before recognising current pregnancy, the smoking cessation period was calculated from the maternal age at birth to the age at quitting smoking. We categorised the number of cigarettes smoked per day as 0 to 5, 6 to 10 and 11 or more; the duration of smoking as 0 to 5 years, 6 to 10 years, and 11 years or more; and the duration of smoking cessation as 0 to 5 years, 6 to 10 years, and 11 years or more. For second-hand smoking, a mother answered during the second (from week 13 to the end of week 26) or third trimester (from week 27 to the end) of pregnancy about how often she had a chance to inhale tobacco smoke at any indoor places by choosing frequency from either almost never or never, once a week, 2–3 times a week, 4–6 times a week, or every day. We have re-categorised it into three categories: seldom, 1–3 times a week, and 4–7 times a week.

The SHS exposure of the children after birth was identified using a self-report questionnaire administered to the mother when the child was 1.5 years old. The mothers answered the question of whether the child is exposed to someone’s cigarette smoke by choosing the options of rarely, sometimes, and often. We have re-categorised the options into two, with (sometimes and often) or without (rarely) smokers near children.

There are potential confounding factors for the association between prenatal tobacco smoke exposure and asthma^3–5^. Therefore, we adjusted the analyses for child sex, maternal history of bronchial asthma, maternal age at birth, pre-pregnancy body mass index (BMI), maternal educational level, older siblings, gestational age at birth and mode of delivery. Additionally, we adjusted for potential confounding factors after birth with the following: attends a childcare facility at 1 year of age and breastfeeding at 1 year of age^21,22^. Maternal history of bronchial asthma, maternal age at birth, pre-pregnancy BMI, maternal educational level and older siblings were collected from mothers via self-administered questionnaires during the first and second/third trimesters of pregnancy. Child sex, gestational week at birth and mode of delivery were extracted from medical record transcripts. Information on childcare facility attendance, and breastfeeding was collected from the self-questionnaire at 1 year of age. The parents answered the question on whether the the child was currently breastfeeding and whether the child was currently attending childcare facility (yes or no).

### Outcome definitions

Information on outcomes was collected using a self-administered questionnaire when the children were 3 years of age. The parents answered the question on whether the child was diagnosed with bronchial asthma by a doctor after the age of 2 years. The outcomes of this study were defined using this questionnaire (yes or no).

### Statistical analyses

The association between smoking exposure and bronchial asthma at 3 years of age was subjected to logistic regression analysis to estimate the crude odds ratio (cOR) and adjusted odds ratio (aOR), adding all the covariates and 95% confidence interval (CI). For multivariate analysis, maternal age, maternal history of bronchial asthma, pre-pregnancy BMI, maternal education, child sex, gestational age, delivery mode, older siblings, attendance to childcare facility at 1 year of age, and breastfeeding at 1 year of age were adjusted. To elucidate the association between prenatal maternal smoking exposure and bronchial asthma at 3 years of age, a logistic regression analysis was performed on a population of children without SHS exposure after birth. To elucidate the involvement of SHS exposure in children after birth and bronchial asthma at 3 years of age, logistic regression analysis was performed by stratifying prenatal maternal smoking status. In multivariate analysis, missing values were excluded from the analysis. All statistical analyses were performed using SPSS version 27. A p-value of 0.05 (two-sided) was considered statistically significant.

## Results

The characteristics of the study participants are shown in Supplementary Table S1. Among the 75,411 mother–child pairs with complete data, 5430 (7.2%) children had bronchial asthma at 3 years of age. Regarding the maternal smoking status before childbirth, 45,248 (60.0%) were categorised as ‘Never’, 18,160 (24.1%) as ‘Previously did, but quit before recognising current pregnancy’, 9301 (12.3%) as ‘Previously did, but quit after finding out about current pregnancy’, and 2702 (3.6%) as ‘Yes, I still smoke’.

Table 1 shows the results of the association between smoking exposure status and bronchial asthma at 3 years of age by univariate and multivariate logistic regression analyses. In the multivariate analysis, children of mothers who sustained smoking during pregnancy had a higher risk of bronchial asthma at 3 years of age than those of mothers who never smoked (aOR 1.34, 95% CI 1.15–1.56). Furthermore, children of mothers who quit smoking before recognising the current pregnancy (aOR 1.09, 95% CI 1.02–1.18) or who quit after finding out about the current pregnancy (aOR 1.11, 95% CI 1.01–1.23) had a higher risk of bronchial asthma at 3 years of age than those of mothers who never smoked. Children of mothers who were frequently exposed to SHS at the home, workplace, or any other indoor places during pregnancy had a higher risk of bronchial asthma at 3 years of age than those of mothers who were seldom exposed to SHS (aOR 1.14, 95% CI 1.04–1.25). Children exposed to SHS after birth had a higher risk of bronchial asthma at 3 years old than unexposed children (aOR 1.12, 95% CI 1.04–1.21).

**Table 1:** Association between smoking exposure and risk of bronchial asthma at the age of 3 years.

	cOR (95% CI)	aOR (95% CI)
Maternal smoking status before birth		
Never	1.00	1.00
Quit before recognising current pregnancy	**1.15 (1.07–1.23)**	**1.09 (1.02–1.18)**
Quit after finding out about current pregnancy	**1.26 (1.16–1.37)**	**1.11 (1.01–1.23)**
Still smoke	**1.82 (1.61–2.06)**	**1.34 (1.15–1.56)**
Partner's smoking status before birth		
Never	1.00	1.00
Quit before recognising current pregnancy	1.05 (0.97–1.14)	1.02 (0.94–1.11)
Quit after finding out about current pregnancy	1.05 (0.88–1.25)	1.00 (0.83–1.21)
Still smoke	**1.21 (1.13–1.29)**	1.04 (0.96–1.12)
Frequency of second-hand smoke exposure during pregnancy		
Seldom	1.00	1.00
1–3 times per week	**1.09 (1.01–1.17)**	1.00 (0.92–1.08)
4–7 times per week	**1.37 (1.27–1.47)**	**1.14 (1.04–1.25)**
Second-hand smoke exposure of the child at the age of 1.5 years		
No	1.00	1.00
Yes	**1.25 (1.17–1.33)**	**1.12 (1.04–1.21)**

cOR, crude odds ratio; aOR, adjusted odds ratio; CI, confidence interval. Boldface indicates statistical significance (p < 0.05).

Adjusted for maternal and partner's smoking status before birth, frequency of second-hand smoke exposure during pregnancy, maternal history of bronchial asthma, maternal age at birth, pre-pregnancy body mass index, maternal educational level, child’s sex, gestational age at birth, mode of delivery, attendance to childcare facility at 1 year of age, breastfeeding at 1 year of age, and older siblings.

To clarify the effects of maternal smoking intensity, the association between maternal smoking intensity (number of cigarettes, duration of smoking) and bronchial asthma at 3 years of age was subjected to logistic regression analysis (Table 2). In children of mothers who sustained smoking during pregnancy, a high number of cigarettes smoked per day (6–10 cigarettes: aOR 1.43, 95% CI 1.14–1.80; ≥ 11 cigarettes: aOR 1.53, 95% CI 1.13–2.06) and a long smoking period (6–10 years: aOR 1.34, 95% CI 1.02–1.77; ≥ 11 yeres: aOR 1.39, 95% CI 1.15–1.68) were associated with an increased risk of bronchial asthma in children at 3 years of age. Furthermore, we investigated the duration of smoking cessation and the risk of bronchial asthma at 3 years of age in children of mothers who quit smoking before recognising current pregnancy (Table 3). The results showed that a maternal smoking cessation period of 11 years or more did not increase the risk of bronchial asthma in the child at 3 years of age (aOR 1.00, 95% CI 0.84–1.19).

**Table 2:** Association between smoking exposure and risk of bronchial asthma at the age of 3 years.

Quit before recognising current pregnancy							
Number of cigarettes smoked per day	Number	cOR (95% CI)	aOR (95% CI)	Duration of smoking (years)	Number	cOR (95% CI)	aOR (95% CI)
Never	45,248	1.00	1.00	Never	45,248	1.00	1.00
0–5	6,022	**1.19 (1.08–1.32)**	**1.13 (1.01–1.26)**	0–5	7,507	**1.21 (1.11–1.33)**	**1.12 (1.01–1.23)**
6–10	6,665	1.10 (0.99–1.21)	1.08 (0.97–1.20)	6–10	6,215	**1.13 (1.02–1.25)**	1.10 (0.99–1.22)
≥ 11	4,717	**1.16 (1.04–1.30)**	1.09 (0.96–1.23)	≥ 11	3,680	1.05 (0.92–1.20)	1.07 (0.93–1.24)
Quit after finding out about current pregnancy							
Number of cigarettes smoked per day	Number	cOR (95% CI)	aOR (95% CI)	Duration of smoking (years)	Number	cOR (95% CI)	aOR (95% CI)
Never	45,248	1.00	1.00	Never	45,248	1.00	1.00
0–5	1,842	**1.24 (1.05–1.47)**	1.12 (0.93–1.36)	0–5	1,315	**1.28 (1.05–1.56)**	1.14 (0.91–1.44)
6–10	3,967	**1.38 (1.24–1.56)**	**1.27 (1.11–1.45)**	6–10	2,875	**1.36 (1.19–1.55)**	1.13 (0.97–1.33)
≥ 11	3,166	**1.15 (1.00–1.32)**	0.99 (0.84–1.17)	≥ 11	4,786	**1.22 (1.09–1.36)**	**1.15 (1.01–1.31)**
Still smoke							
Number of cigarettes smoked per day	Number	cOR (95% CI)	aOR (95% CI)	Duration of smoking (years)	Number	cOR (95% CI)	aOR (95% CI)
Never	45,248	1.00	1.00	Never	45,248	1.00	1.00
0–5	970	**1.62 (1.31–2.00)**	1.20 (0.93–1.54)	0–5	241	**1.71 (1.13–2.57)**	1.20 (0.71–2.05)
6–10	1,115	**1.80 (1.49–2.17)**	**1.43 (1.14–1.80)**	6–10	706	**2.04 (1.62–2.55)**	**1.34 (1.02–1.77)**
≥ 11	559	**2.22 (1.74–2.84)**	**1.53 (1.13–2.06)**	≥ 11	1,696	**1.75 (1.50–2.04)**	**1.39 (1.15–1.68)**

cOR, crude odds ratio; aOR, adjusted odds ratio; CI, confidence interval. Boldface indicates statistical significance (*p* < 0.05). Adjusted for partner's smoking status before birth, frequency of second-hand smoke exposure during pregnancy, maternal history of bronchial asthma, maternal age at birth, pre-pregnancy body mass index, maternal educational level, child’s sex, gestational age at birth, mode of delivery, attendance to childcare facility at 1 year of age, breastfeeding at 1 year of age, and older siblings.

**Table 3:** Association between duration of smoking cessation and the risk of bronchial asthma at the age of 3 in mothers who quit smoking before pregnancy.

Duration after quitting smoking (years)	Number	cOR (95% CI)	aOR (95% CI)
Never	45,248	1.00	1.00
0–5	9328	**1.16 (1.07–1.27)**	**1.10 (1.01–1.21)**
6–10	5777	**1.17 (1.06–1.30)**	**1.14 (1.02–1.27)**
≥ 11	2296	1.03 (0.87–1.22)	1.00 (0.84–1.19)

cOR, crude odds ratio; aOR, adjusted odds ratio; CI, confidence interval. Boldface indicates statistical significance (*p* < 0.05).

Adjusted for partner's smoking status before birth, frequency of second-hand smoke exposure during pregnancy, maternal history of bronchial asthma, maternal age at birth, pre-pregnancy body mass index, maternal educational level, child’s sex, gestational age at birth, mode of delivery, attendance to childcare facility at 1 year of age, breastfeeding at 1 year of age, and older siblings.

To clarify the association between prenatal maternal smoking status and bronchial asthma at 3 years of age, we examined children who were not exposed to SHS after birth (n = 56,212) (Table 4). Children of mothers who sustained smoking during pregnancy had a higher risk of bronchial asthma at 3 years of age than those of mothers who never smoked (aOR 1.40, 95% CI 1.12–1.74). Children of mothers who quit smoking before pregnancy also had a higher risk of bronchial asthma at 3 years of age than those of mothers who never smoked. Children of mothers who quit before recognising current pregnancy (aOR 1.12, 95% CI 1.03–1.22) or who quit after finding out about current pregnancy (aOR 1.15, 95% CI 1.02–1.30) also had a higher risk of bronchial asthma at 3 years of age than those of mothers who never smoked.

**Table 4:** Association between prenatal maternal smoking status and risk of bronchial asthma at the age of 3 years in participants without postnatal second-hand smoke exposure.

	cOR (95% CI)	aOR (95% CI)
Maternal smoking status before birth		
Never	1.00	1.00
Quit before recognising current pregnancy	**1.17 (1.08–1.26)**	**1.12 (1.03–1.22)**
Quit after finding out current pregnancy	**1.25 (1.12–1.39)**	**1.15 (1.02–1.30)**
Still smoke	**1.93 (1.60–2.32)**	**1.40 (1.12–1.74)**

cOR, crude odds ratio; aOR, adjusted odds ratio; CI, confidence interval. Boldface indicates statistical significance (*p* < 0.05).

Adjusted for partner’s smoking status before birth, frequency of second-hand smoke exposure during pregnancy, maternal history of bronchial asthma, maternal age at birth, pre-pregnancy body mass index, maternal educational level, child’s sex, gestational age at birth, mode of delivery, attendance to childcare facility at 1 year of age, breastfeeding at 1 year of age, and older siblings.

Subsequently, to clarify the effects of postnatal SHS exposure, a logistic regression analysis was performed by stratifying the maternal smoking status before childbirth (Table S2). In a group of mothers who had never smoked before childbirth, children exposed to SHS after birth had a higher risk of bronchial asthma at 3 years of age than unexposed children (aOR 1.23, 95% CI 1.11–1.36). In contrast, there was no significant difference in the risk of bronchial asthma at 3 years of age among children exposed to SHS after birth and unexposed children in any group of mothers who smoked before childbirth.

## Discussion

In a large birth cohort study, we found strong evidence that children of mothers who sustained smoking during pregnancy had an increased risk of bronchial asthma at 3 years of age even when adjusted for postnatal covariates such as attendance to nursery school, and breastfeeding. Additionally, we found that children of mothers who had smoked in early pregnancy and before pregnancy also had a significantly increased risk of bronchial asthma at 3 years of age. Evaluations at baseline and 1 year after study inception showed the representativeness of the study population to the general population in Japan^20,23^, suggesting that the results of this study may be generalised.

Several cohort studies have previously reported that maternal smoking during pregnancy increases the risk of asthma in children. In a meta-analysis of eight European birth cohorts^9^, maternal smoking during pregnancy is associated with asthma at preschool age, with an aOR of 1.65 (95% CI 1.18–2.31). In a large-scale cohort study (n = 60,254) in Finland^24^, both maternal light (< 10 cigarettes per day) and heavy (≥ 10 cigarettes per day) smoking during pregnancy increased the risk of asthma by 7 years of age, with odds ratios of 1.23 (95% CI 1.07–1.42) and 1.35 (95% CI 1.13–1.62), respectively, compared with never smoking. Our large birth cohort study found that the risk of bronchial asthma at 3 years of age increases not only with the number of cigarettes smoked per day but also with the duration of smoking. The association between pre- and postnatal SHS exposure and the development of asthma in children is controversial^13,16,25^. Our results found that children of mothers who were frequently exposed to SHS at the home, workplace, or any other indoor places during pregnancy had an increased risk of bronchial asthma at 3 years of age. Additionally, postnatal SHS exposure significantly increased the risk of bronchial asthma at 3 years of age even after adjusting for other covariates such as prenatal smoking exposure.

We found that prenatal smoking exposure was associated with an increased risk of bronchial asthma at 3 years of age, even in the absence of postnatal smoking exposure. The concept of Developmental Origins of Health and Disease (DOHaD) is considered a molecular mechanism that links prenatal smoking exposure and bronchial asthma. DOHaD evaluates how the early-life environment can impact the risk of non-communicable diseases from childhood to adulthood^26^. Epigenetic modifications such as DNA methylation are considered the molecular mechanism of DOHaD^27–29^. Maternal smoking during pregnancy has been shown to alter umbilical cord blood DNA methylation in various genes such as aryl-hydrocarbon receptor repressor (*AHRR*), cytochrome P450 family 1 subfamily A member 1 (*CYP1A1*), growth factor-independent 1 transcriptional repressor (*GFI1*), and myosin IG (*MYO1G*)^30,31^. It has been suggested that this DNA methylation change is maintained throughout the life course^32,33^. Neophytou et al. showed that it suggests a potential mediation of *AHRR* methylation in the association between maternal smoking during pregnancy and asthma in Latino children^34^. Gao also showed a synergistic effect of prenatal maternal smoking and AXL receptor tyrosine kinase (AXL) methylation on the risk of childhood bronchitis symptoms^35^. Thus, although methylation of several genes has been reported to be associated with maternal smoking exposure and the risk of childhood asthma, it is necessary to elucidate the molecular mechanisms underlying the development of asthma in the future.

To the best of our knowledge, the present study is the first report showing that children of mothers who quit smoking before recognising the current pregnancy or quit after finding out about the current pregnancy also had a significantly increased risk of bronchial asthma at 3 years of age. It has been suggested that pre-conception exposure to tobacco, chemicals, and stress can cause epigenomic changes in germ cells, adversely affecting the health of the next generation^36^. Pre-pregnancy female mouse exposure to cyclophosphamide, a widely used drug in the treatment of breast cancer, has been shown to alter DNA methylation in F1 and F2 mouse oocytes^37^. Wu et al. recently reported that prenatal paternal smoking exposure increased DNA methylation of immune-related genes, such as *LMO2* and *IL-10,* and correlated with the development of asthma in children^38^. Furthermore, a three-generation cohort study suggests that maternal smoking before conception is associated with an increased risk of childhood asthma^39,40^. Therefore, it is suggested that epigenetic changes due to the parental smoking history may be transmitted on to the next generation through germ lines even if they are not smoking during pregnancy. It is important for mothers to quit smoking as soon as possible, as we have shown that the longer the mother quits smoking, the lower the risk of bronchial asthma in the child at 3 years of age.

This study has several limitations. First, the smoking status of parents was self-reported and may be misclassified. Our results show that the prevalence of smoking among pregnant women is 3.6%, which is similar to other studies. However, it has been suggested that the actual smoking prevalence among pregnant women is much higher. Using JECS, Nishihama et al. found a difference between the prevalence of smoking in pregnant women based on questionnaires (4.6%) and the prevalence of smoking in pregnant women based on urinary cotinine levels (8%)^41^. They also reported that 78% of passive smokers might be misclassified as non-smokers during pregnancy. Analysis using cut-offs based on urinary cotinine levels in this study did not show a significant association between SHS exposure during pregnancy and an increased risk of asthma at 3 years of age (Supplementary Table S3). Therefore, there is a risk of both under- and over reporting of the diagnosis in maternal smoking exposure during pregnancy. Second, smoking prevalence among pregnant women in the study participants was lower than baseline, suggesting that selection bias may have led to an underestimation of asthma risk at 3 years of age. Third, since the study participants were recruited in early pregnancy, there is a possibility that recall bias may have influenced the maternal smoking status before pregnancy. Fourth, the effects of SHS exposure on children after birth are conducted using a 1.5-year-old questionnaire. Passive smoking exposure of postnatal children was investigated using a questionnaire at the age of 1.5 years. It is unknown how long a child was exposed to SHS from birth to age 3 years. Fifth, doctor-diagnosed asthma was defined based on a self-administered questionnaire to caregivers. As it is difficult to confirm the diagnosis of asthma in infants and preschool children^42^, some outcomes of this study may have been misclassified. Sixth, missing covariate data can cause bias in multivariate analysis. However, the result of the sensitivity analysis using the dataset in which the missing value was substituted by the multiple imputation method was not different from the result of the dataset in which the missing value was deleted (Supplementary Table S3). Therefore, the results of this study are robust. Finally, we have adjusted for the essential confounding factors identified in previous studies, but observational studies cannot exclude the possibility of residual confounding.

## Conclusions

Our results from a large birth cohort study strengthened the existing evidence that maternal smoking throughout pregnancy increases the risk of childhood bronchial asthma, further identifying new features in this association. We have suggested that the effects of maternal smoking even before pregnancy may increase the risk of asthma in children. Therefore, it is important for both parents to quit smoking early to reduce the risk of future health hazards to their children.

## Supplementary Information

Supplementary Information.

## Data Availability

Data are unsuitable for public deposition due to ethical restrictions and legal framework of Japan. It is prohibited by the Act on the Protection of Personal Information (Act No. 57 of 30 May 2003, amendment on 9 September 2015) to publicly deposit the data containing personal information. Ethical Guidelines for Medical and Health Research Involving Human Subjects enforced by the Japan Ministry of Education, Culture, Sports, Science and Technology and the Ministry of Health, Labour and Welfare also restricts the open sharing of the epidemiologic data. All inquiries about access to data should be sent to: jecs-en@nies.go.jp. The person responsible for handling enquiries sent to this e-mail address is Dr Shoji F.Nakayama, JECS Programme Office, National Institute for Environmental Studies.
