# Supplementary material for: Maternal smoking status before and during pregnancy and bronchial asthma at 3 years of age: a prospective cohort study
Source: Sci Rep. 2023 Feb 24;13:3234. doi: 10.1038/s41598-023-30304-9 (PMC9958124; doi:10.1038/s41598-023-30304-9)
Supplement: Supplementary file 1 — Supplementary Information. [file 41598_2023_30304_MOESM1_ESM.docx]

**Maternal smoking status before and during pregnancy and bronchial asthma at 3 years of age: a prospective cohort study**

Kunio Miyake^1^*, Megumi Kushima^2^, Ryoji Shinohara^2^, Sayaka Horiuchi^2^, Sanae Otawa^2^, Yuka Akiyama^1^, Tadao Ooka^1^, Reiji Kojima^1^, Hiroshi Yokomichi^1^, Zentaro Yamagata^1,2^ and the Japan Environment and Children's Study Group^3^

**Supplementary Methods**

The characteristics of the study participants are shown in Supplementary Table S1.

In the JECS, Nishihama et al. recently reported cut-off values for passive and active smokers using urinary cotinine concentration in the second or third trimester of pregnancy [37]. The cut-off values that distinguish non-smokers from passive smokers and passive smokers from active smokers were 0.31 and 36.8 μg/g-creatinine, respectively. We categorised non-smokers, and passive and active smokers based on this cut-off value. Table S2 shows the association between prenatal smoking exposure based on the cut-off of urinary cotinine levels and the risk of bronchial asthma at 3 years of age.

We used SPSS version 27 multiple imputation analysis to create a dataset pooled from the 20 imputed datasets and performed logistic regression analysis. Table S3 shows the association between smoking exposure status and bronchial asthma at 3 years of age.

Table S1. Characteristics of the study population

|  |  | **Maternal smoking status before birth** | | | |
| --- | --- | --- | --- | --- | --- |
|  | All  75,411 (100) | Never 45,248 (60.0) | Quit before recognising current pregnancy  18,160 (24.1) | Quit after finding out about current pregnancy  9,301 (12.3) | Still smoke  2,702 (3.6) |
| **Partner’s smoking status before birth** |  |  |  |  |  |
| Never | 21,503 (28.9) | 16,808 (22.6) | 3,743 (5.0) | 815 (1.1) | 137 (0.2) |
| Quit before recognising current pregnancy | 18,019 (24.2) | 11,723 (15.7) | 5,452 (7.3) | 693 (0.9) | 151 (0.2) |
| Quit after finding out about current pregnancy | 2,136 (2.9) | 891 (1.2) | 520 (0.7) | 705 (0.9) | 20 (0.0) |
| Still smoke | 32,810 (44.1) | 15,394 (20.7) | 8,221 (11.0) | 6,897 (9.3) | 2,298 (3.1) |
| **Frequency of second-hand smoke exposure during pregnancy** |  |  |  |  |  |
| Seldom | 48,573 (64.6) | 32,717 (43.5) | 11,615 (15.4) | 3,854 (5.1) | 387 (0.5) |
| 1–3 times per week | 14,682 (19.5) | 7,892 (10.5) | 3,847 (5.1) | 2,481 (3.3) | 462 (0.6) |
| 4–7 times per week | 11,991 (15.9) | 4,554 (6.1) | 2,660 (3.5) | 2,936 (3.9) | 1,841 (2.4) |
| **Maternal history of bronchial asthma** |  |  |  |  |  |
| No | 67,205 (89.5) | 40,796 (54.3) | 16,062 (21.4) | 8,061 (10.7) | 2,286 (3.0) |
| Yes | 7,923 (10.5) | 4,282 (5.7) | 2,029 (2.7) | 1,210 (1.6) | 402 (0.5) |
| **Maternal age at birth (years)** |  |  |  |  |  |
| > 30 | 26,623 (35.3) | 15,328 (20.3) | 5,422 (7.2) | 4,631 (6.1) | 1,242 (1.6) |
| ≥ 30 and < 40 | 45,162 (59.9) | 27,673 (36.7) | 11,754 (15.6) | 4,398 (5.8) | 1,337 (1.8) |
| ≥ 40 | 3,625 (4.8) | 2,247 (3.0) | 983 (1.3) | 272 (0.4) | 123 (0.2) |
| **Pre-pregnancy BMI** |  |  |  |  |  |
| < 18.5 | 12,227 (16.2) | 7,531 (10.0) | 2,544 (3.4) | 1,642 (2.2) | 510 (0.7) |
| ≥ 18.5 and < 25 | 55,664 (73.9) | 33,755 (44.8) | 13,566 (18.0) | 6,533 (8.7) | 1,810 (2.4) |
| ≥ 25 | 7,479 (9.9) | 3,943 (5.2) | 2,037 (2.7) | 1,118 (1.5) | 381 (0.5) |
| **Maternal educational level (years)** |  |  |  |  |  |
| ≤ 12 | 25,051 (33.4) | 10,968 (14.6) | 7,028 (9.4) | 5,139 (6.8) | 1,916 (2.6) |
| > 12 | 50,003 (66.6) | 34,086 (45.4) | 11,042 (14.7) | 4,110 (5.5) | 765 (1.0) |
| **Gestational weeks at birth (weeks)** |  |  |  |  |  |
| ≥ 37 | 71,345 (94.8) | 42,872 (57.0) | 17,141 (22.8) | 8,814 (11.7) | 2,518 (3.3) |
| < 37 | 3,923 (5.2) | 2,300 (3.1) | 979 (1.3) | 465 (0.6) | 179 (0.2) |
| **Child’s sex** |  |  |  |  |  |
| Male | 38,573 (51.2) | 23,146 (30.7) | 9,236 (12.2) | 4,779 (6.3) | 1,412 (1.9) |
| Female | 36,838 (48.8) | 22,102 (29.3) | 8,924 (11.8) | 4,522 (6.0) | 1,290 (1.7) |
| **Mode of delivery** |  |  |  |  |  |
| Vaginal | 60,347 (80.4) | 36,471 (48.6) | 14,337 (19.1) | 7,408 (9.9) | 2,131 (2.8) |
| Caesarean section | 14,726 (19.6) | 8,581 (11.4) | 3,734 (5.0) | 1,849 (2.5) | 562 (0.7) |
| **Attendance to childcare facility at 1 year of age** |  |  |  |  |  |
| No | 55,643 (75.6) | 34,094 (46.3) | 13,273 (18.0) | 6,557 (8.9) | 1,719 (2.3) |
| Yes | 17,939 (24.4) | 10,283 (14.0) | 4,454 (6.1) | 2,386 (3.2) | 816 (1.1) |
| **Breastfeeding at 1 year of age** |  |  |  |  |  |
| No | 28,401 (38.7) | 15,405 (21.0) | 6,848 (9.3) | 4,561 (6.2) | 1,587 (2.2) |
| Yes | 45,036 (61.3) | 28,913 (39.4) | 10,860 (14.8) | 4,331 (5.9) | 932 (1.3) |
| **Older siblings** |  |  |  |  |  |
| No | 35,195 (46.8) | 21,665 (28.8) | 7,018 (9.3) | 5,399 (7.2) | 1,113 (1.5) |
| Yes | 39,933 (53.2) | 23,413 (31.2) | 11,073 (14.7) | 3,872 (5.2) | 1,575 (2.1) |
| **Second-hand smoke exposure of the child at the age of 1.5 years** |  |  |  |  |  |
| No | 56,212 (77.0) | 36,106 (49.4) | 13,296 (18.2) | 5,666 (7.8) | 1,144 (1.6) |
| Yes | 16,812 (23.0) | 7,915 (10.8) | 4,298 (5.9) | 3,222 (4.4) | 1,377 (1.9) |
| **Bronchial asthma at the age of 3 years** |  |  |  |  |  |
| No | 69,981 (92.8) | 42,253 (56.0) | 16,795 (22.3) | 8,540 (11.3) | 2,393 (3.2) |
| Yes | 5,430 (7.2) | 2,995 (4.0) | 1,365 (1.8) | 761 (1.0) | 309 (0.4) |

n (%)

Table S2. Association between postnatal second-hand smoke exposure and risk of bronchial asthma at the age of 3 years

| Maternal smoking status before birth | Postnatal SHS exposure | cOR (95% CI) | aOR (95% CI) |
| --- | --- | --- | --- |
| Never | No | 1.00 | 1.00 |
|  | Yes | **1.26 (1.15–1.38)** | **1.23 (1.11–1.36)** |
| Quit before recognising current pregnancy | No | 1.00 | 1.00 |
|  | Yes | 1.13 (0.99–1.29) | 1.04 (0.90–1.20) |
| Quit after finding out current pregnancy | No | 1.00 | 1.00 |
|  | Yes | 1.11 (0.95–1.30) | 1.01 (0.85–1.21) |
| Still smoke | No | 1.00 | 1.00 |
|  | Yes | 0.97 (0.76–1.25) | 1.01 (0.76–1.35) |

cOR, crude odds ratio; aOR, adjusted odds ratio; CI, confidence interval. Boldface indicates statistical significance (p < 0.05).

Adjusted for partner's smoking status before birth, frequency of second-hand smoke exposure during pregnancy, maternal history of bronchial asthma, maternal age at birth, pre-pregnancy body mass index, maternal educational level, child’s sex, gestational age at birth, mode of delivery, attendance to childcare facility at 1 year of age, breastfeeding at 1 year of age, and older siblings.

Table S3. Association between maternal cotinine levels at the second or third trimester of pregnancy and risk of bronchial asthma at the age of 3

| Cotinine (μg/g-creatinine) | n (%) | cOR (95% CI) | aOR (95% CI) |
| --- | --- | --- | --- |
| Non-smokers (< 0.31) | 42,671 (56.6) | 1.00 | 1.00 |
| Passive smokers (0.31–36.8) | 25,235 (33.5) | **1.09 (1.03–1.16)** | 1.02 (0.96**–**1.09) |
| Active smokers (> 36.8) | 4,793 (6.4) | **1.55 (1.40–1.71)** | **1.27 (1.13–1.42)** |

cOR, crude odds ratio; aOR, adjusted odds ratio; CI, confidence interval. Boldface indicates statistical significance (p < 0.05).

Adjusted for maternal history of bronchial asthma, maternal age at birth, pre-pregnancy body mass index, maternal education level, child’s sex, gestational age at birth, mode of delivery, attendance to childcare facility by 1 year of age, older siblings, and child’s exposure to second-hand smoke at the age of one and a half.

Table S4. Association between smoking exposure and risk of bronchial asthma at the age of 3 years by logistic regression analysis in the imputed dataset

|  | cOR (95% CI) | aOR (95% CI) |
| --- | --- | --- |
| **Maternal smoking status before birth** |  |  |
| Never | 1.00 | 1.00 |
| Quit before recognising current pregnancy | **1.16 (1.08–1.25)** | **1.09 (1.01–1.18)** |
| Quit after finding out about current pregnancy | **1.30 (1.18–1.42)** | **1.14 (1.03–1.27)** |
| Still smoke | **1.93 (1.61–2.31)** | **1.45 (1.22–1.71)** |
| **Partner's smoking status before birth** |  |  |
| Never | 1.00 | 1.00 |
| Quit before recognising current pregnancy | 1.06 (0.98–1.15) | 1.04 (0.96–1.12) |
| Quit after finding out about current pregnancy | 1.05 (0.90–1.23) | 0.99 (0.84–1.16) |
| Still smoke | **1.25 (1.18–1.34)** | 1.03 (0.95–1.11) |
| **Frequency of second-hand smoke exposure during pregnancy** |  |  |
| Seldom | 1.00 | 1.00 |
| 1–3 times per week | **1.10 (1.03–1.18)** | 1.00 (0.93–1.08) |
| 4–7 times per week | **1.46 (1.34–1.59)** | **1.14 (1.04–1.25)** |
| **Second-hand smoke exposure of the child at the age of one and a half years** |  |  |
| No | 1.00 | 1.00 |
| Yes | **1.31 (1.20–1.43)** | 1.11 (0.99–1.24) |

cOR, crude odds ratio; aOR, adjusted odds ratio; CI, confidence interval. Boldface indicates statistical significance (p < 0.05).

Adjusted for maternal and partner's smoking status before birth, frequency of second-hand smoke exposure during pregnancy, maternal history of bronchial asthma, maternal age at birth, pre-pregnancy body mass index, maternal educational level, child’s sex, gestational age at birth, mode of delivery, attendance to childcare facility at 1 year of age, breastfeeding at 1 year of age, and older siblings.
